# Comparison between Sigma metrics in four accredited Egyptian medical laboratories in some biochemical tests: an initiative towards sigma calculation harmonization

**DOI:** 10.11613/BM.2018.020711

**Published:** 2018-06-15

**Authors:** Rania El Sharkawy, Sten Westgard, Ahmed M Awad, AbdelKarem Omneya I Ahmed, El Hadidi Iman, Ahmed Gaballah, Eman Shaheen

**Affiliations:** 1 Medical Research Institute, Alexandria University, Alexandria, Egypt; 2 Westgard QC Inc., Madison, USA; 3 Ain Shams University, Cairo, Egypt; 4 Zagazig University, Zagazig, Egypt; 5 Private laboratory, Helwan University, Alexandria, Egypt

**Keywords:** sigma, bias, coefficient of variation, harmonization, total allowable error

## Abstract

**Introduction:**

Analytical quality is an essential requirement for best practice in any medical laboratory. Lack of a harmonized approach for sigma calculation is considered an obstacle in the objective comparability of analytical performance among laboratories adopting sigma metrics. It is urgently needed that all laboratory professionals interested in the analytical quality to work hard towards harmonization protocol for sigma calculation in order to properly select their analytical goals. This study aims at harmonization of Sigma metrics calculation in four accredited Egyptian laboratories.

**Materials and methods:**

This observational cross sectional study compared the sigma levels for certain biochemical parameters in the four participating laboratories.

**Results:**

Coefficient of variation (CV) and bias were determined for some biochemical analytes, data assayed by different automated analysers in the four different accredited laboratories. The sigma level for the four medical laboratories was calculated for each biomedical parameter with changed sigma level after total allowable error (Tea) unification among participating laboratories.

**Conclusion:**

Each laboratory should select the TEa goal based on clear standardized criteria of selection without any subjective preferences as either under or over estimation of Sigma metrics will affect the patient centred care negatively if laboratories use quality control procedures wrongly based on incorrect Sigma metrics calculation with subsequent misleading medical decisions.

## Introduction

Analytical quality is an essential requirement for best practice in any medical laboratory. Patient-centred care, the main target of medical laboratories, depends on the key concepts of internal quality control (IQC) which was established by Levey and Jennings followed by the interpretative rules published by Westgard and colleagues and external quality assurance (EQA) programs, which established in the late nineties as a complementary pillar to IQC, provide a tool of peer comparison (*1*-*5*). Consequently, the minimization of analytical imprecision reflected as random errors *via* proper IQC plans and minimization of analytical bias seen as systematic errors through EQA programs are considered fundamental tools for any quality management system in laboratory medicine (*6*).

Quality decision specifications based on laboratory performance characteristics (bias and imprecision) were recommended many years ago as a mechanism to support quality in the medical laboratories. It is now necessary to determine how the performance of a measurement procedure relates to the medical requirements for interpreting results in order to determine the frequency to measure and evaluate quality control (QC) samples and results (*7*, *8*). Sigma metrics (SM) have been used to assess quality in a quantitative manner. There are two different methodologies for assessing process performance in terms of Sigma metrics. The first method depends on counting the defects or errors which are expressed as defects *per* million (DPM); the DPM are subsequently converted to a SM scale of 0 to 6, with 6 being world class (3.4 defects *per* million) and 3 being the minimum level of performance (about 66,800 defects *per* million) (*9*).

The second method depends mainly on measuring the variation of the measurement process to predict its performance and evaluate how well a measurement procedure performs using the two pillars of performance characteristics (bias and precision) and the total allowable error (TEa) (*10*). The goal is to seek for 6-sigma (world class) quality, with the common minimum level of acceptable quality broadly considered to be 3-sigma (*11*).

The use of SM offers many advantages to laboratories as it helps in determining their IQC frequency; thus avoiding repeated IQC testing during periods of stable performance, consequently minimizing unnecessary costs and human-hour wastage. In addition, it facilitates the comparison of the same assay performance across multiple systems (*9*, *12*, *13*).

To the best of our knowledge, this study represents the first study that tackles the variation in Sigma metrics calculation among accredited laboratories in Egypt. Since most of the laboratories are calculating the sigma and comparing the results while using different methods of calculating the sigma elements (bias and CV) as well as selecting suitable TEa, this variation might affect the comparability of analytical performance though they are all accredited. Also, it sheds the light over some key points in Sigma metrics calculation that allows laboratorians to make use of such valuable tool for assessment of method performance in a more objective manner.

This study aims at harmonization of Sigma metrics calculation following a standardized protocol in order to improve its utility for evaluation of the performance among accredited laboratories which is consequently reflected on the patient centred care in addition to facilitating comparability of sigma values in a more objective methodology.

## Materials and methods

### Study design

The current study is an observational cross sectional study which was conducted after approval of the Research ethical committees in each university and the whole study design was approved by the Medical Research Institute, ethics committee. The presented data are from four Egyptian International Organization for standardization (ISO) 15189:2012 accredited medical laboratories: Chemical Pathology department Medical Research Institute, Alexandria University (MRI laboratory) a hospital laboratory in Alexandria governorate, Zagazig University hospital laboratory located in Zagazig governorate, Ain Shams University hospital laboratory located in Cairo governorate and a private laboratory in Alexandria governorate.

### Methods

Coefficient of variation (CV, from IQC records) and bias (from proficiency testing data) were determined for some biochemical analytes, data assayed by different automated analysers in the four different accredited laboratories then Sigma metrics calculation was performed.

Estimated parameters were glucose (Glc), urea, creatinine (CREA), uric acid (UA), cholesterol (CHOL), triglycerides (Tg), albumin (Alb) bilirubin, direct (BD), bilirubin, total (BT), total protein (TP), calcium (Ca), inorganic phosphates (Phos), magnesium (Mg) and potassium (K). Moreover, the following enzyme activities were measured: alanine aminotransferase (ALT), alkaline phosphatase (ALP), lactate dehydrogenase (LD) and gamma-glutamyltransferase (GGT). As well as two immunoassay parameters were evaluated: alpha fetoprotein (AFP) and carcinoembryonic antigen (CEA). Some parameters were not calculated by the four laboratories and presented in the current study to show the effect of different TEa on sigma calculation to emphasize on the idea of need for harmonization of the current sigma calculation.

The instruments used by the four laboratories: Olympus AU 400 (Beckman Coulter International SA, Nyon, Switzerland) was used in MRI laboratory where most of biochemical reagents were dedicated Beckman Coulter except bilirubin (Spectra, Cairo, Egypt), and creatinine (Randox, Antrim, United Kingdom). Immunoassay parameters were assayed on Immulite 1000 (Siemens Healthineers GmbH, Erlangen, Germany), while AVL 9180 (Roche Diagnostics GmbH, Mannheim, Germany) was used to assay the potassium. In Zagazig University hospital laboratory Cobas 8000 modular system (Roche Diagnostics GmbH, Mannheim, Germany) was used to assay both biochemical and immunoassay parameters while AVL 9180 (Roche Diagnostics GmbH, Mannheim, German) was used to assay the potassium. As for Ain Shams University hospital laboratory; Olympus AU 480 with Beckman Coulter dedicated reagents (Beckman Coulter International SA, Nyon, Switzerland) was used and AVL 9180 (Roche Diagnostics GmbH, Mannheim, Germany) was used to assay the potassium. The data collected from Ain Shams University hospital laboratory lacked of the immunoassay parameters. Finally the private laboratory used Cobas c501 (Roche Diagnostics GmbH, Mannheim, Germany) to assay biochemical parameters.

Internal QC data was extracted from the analysers records from January till May 2016 (130 QC run during 130 working days / one run *per* day). Control materials were run before each analytical run. Each laboratory has its customized internal quality control and calibrations protocol which was done according to each laboratory internal quality control policies and procedure. Each laboratory selected the TEa according to the current analytical laboratory performance.

Internal quality control (IQC) data (same lot for each laboratory and level 1 QC values determined by manufacturers) were used by the four laboratories to determine each parameter CV after exclusion of outliers (QC observations that violate 1_3S_ rule). External quality assurance (EQA) data were used by the four laboratories to determine bias for each analyte. The results of EQA samples for at least 3 months were included, the EQA programs used by the four laboratories were not accuracy based and the mean of comparator group is considered as consensus group peer data. The mean of comparators selected according to each laboratory method and instrument, so there were no real true values used in the current study by any of the participating laboratories to determine the bias of the studied biochemical parameters.

The participating laboratories assayed the BioRad monthly program as external quality assessment scheme which consisted of twelve monthly samples in each cycle. According to the manufacturer, the total number of samples for the entire cycle was provided at the same time. All submitted results for each analyte are grouped according to comparators (peer, method, and mode/all results) then an ISO 13528 robust statistical analysis was performed (*14*).

The approach used in the current study to calculate the Sigma metrics relied on method performance measurement. For laboratory measurements, the Sigma metrics is calculated by the following formula (*8*): Sigma metrics = (TEa – bias observed) / CV (coefficient of variation) observed. The studied parameters were sorted into 6 categories; world class performance (SM = 6 or more), excellent performance (SM = 5-6), good performance (SM = 4-5), marginal performance (SM = 3-4), poor performance (SM = 2-3) and unacceptable performance (SM is less than 2).

The CV is estimated from the QC data as previously described. It is critically important that the estimate of CV is done using QC data that represent all or most components of variability that occur over an extended time period. A CV that represents stable measurement performance can usually be estimated from the cumulative standard deviation (SD) over a 6 to 12-month period for a single lot of QC material (*8*). It is noteworthy to state that there was no unified protocol used in the current study to estimate either CV or mean % bias of the studied parameters and each laboratory calculate these performance characteristics according to each laboratory policy.

Two Sigma metrics were calculated for each parameter using 2 different CVs that were obtained from 2 levels IQC data.

The harmonization protocol (Annex 1) is a novel protocol that was suggested by the working group of the current study. It aims at checking most of the key points that are considered as potential sources of variability in Sigma metrics calculation.

### Data presentation

The formula CV = (Standard deviation / mean) x 100 has been used for the calculation of coefficient of variation. Bias (%) calculation of single PT measurement using Bio-Rad EQAS programs was calculated using [(mean of all laboratories using same instrument/method – laboratory mean) / mean of all laboratories using same instrument and method] x 100. Mean bias (%) was calculated through the sum of all % bias of PT values of specific parameter / number of PT values. The Sigma metrics was calculated as (TEa – Bias observed / CV observed).

## Results

Table 1 shows the performance characteristics of the parameters from Medical Research Institute, Alexandria University, Chemical Pathology Laboratory; Sigma metrics were calculated using the total allowable errors from the different sources as shown. Among the assayed parameters total bilirubin had the highest sigma (10.5) while magnesium had the lowest sigma value (- 0.7).

**Table 1:** Chemical Pathology department, Medical Research Institute, Alexandria University sigma metrics calculation

**Parameters**	**TEa source**	**TEa**	**Bias (%)**	** CV (%)**	**Sigma**
AFP	BV minimal	32.80	9.3	6.7	3.5
ALT	BV desirable	27.48	6.7	3.4	6.1
Alb	BV desirable	4.07	0.2	1.9	2.0
ALP	BV desirable	12.04	2.3	6.9	1.4
BD	BV desirable	44.50	5.6	7.5	5.2
BT	BV desirable	26.94	0.0	2.5	10.5
Calcium	BV minimal	3.82	2.0	2.3	0.8
CEA	RiliBÄK	24.00	1.3	11.4	2.0
Cholesterol	BV desirable	9.01	6.1	2.6	1.1
Creatinine	BV desirable	8.87	5.5	7.5	0.4
Glucose	BV desirable	6.96	1.1	1.4	4.2
LD	BV desirable	11.35	0.5	4.7	2.3
Magnesium	BV minimal	7.20	10.0	4.3	- 0.7
Phosphorous	BV desirable	10.11	5.1	3.5	1.2
Potassium	BV desirable	5.61	2.1	5.1	0.7
Total protein	BV desirable	3.63	0.2	3.3	1.0
Triglycerides	BV desirable	25.99	1.5	3.1	7.9
Uric acid	BV desirable	11.97	0.7	1.9	5.9
Urea	BV desirable	15.55	1.9	1.9	7.2
GGT	BV desirable	22.11	1.	11.0	1.9
TEa – total allowable error. CV – coefficient of variation. BV – biological variation. RiliBÄK - guidelines of the German medical association for the quality assurance of laboratory medical examinations. AFP - alpha fetoprotein. ALT - alanine aminotransferase. Alb – albumin. ALP - alkaline phosphatase. BD – billirubin, direct. BT- bilirubin, total. CEA - carcinoembryonic antigen. LD - lactate dehydrogenase. GGT - gamma glutamyltransferase.					

Table 2 shows the performance characteristics of the parameters assayed in Zagazig University Hospital laboratory. Sigma metrics were calculated using the total allowable errors from the different sources as shown in the table. Triglycerides and direct bilirubin had the highest sigma (10.7) while calcium had the lowest sigma value (0.7) among the assayed parameters.

**Table 2:** Zagazig University Hospital laboratory Sigma metrics calculation

**Parameters**	**TEa source**	**TEa**	**Bias (%)**	**CV (%)**	**Sigma**
AFP	BV desirable	21.87	4.2	5.8	3.0
ALT	BV desirable	27.48	8.7	4.6	4.1
Alb	CLIA	10.00	0.6	2.0	4.6
ALP	CLIA	30.00	2.7	5.0	5.4
BD	BV desirable	44.50	2.7	3.9	10.7
BT	BV desirable	26.94	6.4	3.3	6.2
Calcium	BV Minimal	3.82	2.3	1.9	0.7
Cholesterol	BV desirable	9.01	3.2	1.6	3.3
Creatinine	BV desirable	8.87	1.9	3.8	1.8
Glucose	CLIA	10.00	1.7	2.1	3.9
LD	CLIA	20.00	1.9	3.4	5.3
Magnesium	BV Minimal	7.21	0.5	4.1	1.6
Phosphorous	BV Minimal	15.16	1.3	4.0	3.4
Potassium	BV desirable	5.61	1.1	2.4	1.8
Total protein	CLIA	10.00	1.7	1.9	4.3
Triglycerides	BV desirable	25.99	1.4	2.3	10.7
Uric acid	BV desirable	11.97	1.0	2.4	4.6
Urea	BV desirable	15.55	0.0	4.0	3.9
GGT	BV desirable	22.11	2.3	3.2	6.2
TEa – total allowable error. CV – coefficient of variation. BV – biological variation. CLIA - Clinical Laboratory Improvement Amendments. AFP - alpha fetoprotein. ALT - alanine aminotransferase. Alb – albumin. ALP - alkaline phosphatase. BD – billirubin, direct. BT- bilirubin, total. CEA - carcinoembryonic antigen. LD - lactate dehydrogenase. GGT - gamma glutamyltransferase.					

Table 3 shows the performance characteristics of the parameters from Ain Shams University hospital laboratory; Sigma metrics were calculated using the total allowable errors from the different sources as shown in the table. Among the assayed parameters glucose had the highest sigma (4.9) while albumin had the lowest sigma value (0.4).

**Table 3:** Ain Shams University hospital laboratory Sigma metrics calculation

**Parameters**	**TEa source**	**TEa**	**Bias (%)**	**CV (%)**	**Sigma**
ALT	CLIA	20.00	5.9	6.7	2.1
Alb	CLIA	10.0	7.2	7.2	0.4
ALP	CLIA	30.00	3.9	8.0	3.3
BD	BV desirable	44.50	8.9	9.4	3.8
BT	CLIA	20.00	2.1	6.6	2.7
Calcium	CLIA	16.53	0.5	6.0	2.7
Cholesterol	CLIA	10.00	1.7	6.3	1.3
Creatinine	CLIA	15.00	0.6	7.9	1.8
Glucose	CLIA	10.00	1.1	1.8	4.9
Magnesium	CLIA	25.00	8.0	8.4	2.0
Potassium	CLIA	8.33	2.1	8.0	0.8
Total protein	CLIA	10.00	1.4	6.9	1.2
Uric acid	CLIA	17.00	3.0	4.6	3.0
Urea	CLIA	9.00	6.3	6.0	0.5
GGT	CLIA	26.90	4.6	10.4	2.1
TEa – total allowable error. CV – coefficient of variation. BV – biological variation. CLIA - Clinical Laboratory Improvement Amendments. AFP - alpha fetoprotein. ALT - alanine aminotransferase. Alb – albumin. ALP - alkaline phosphatase. BD – billirubin, direct. BT- bilirubin, total. CEA - carcinoembryonic antigen. LD - lactate dehydrogenase. GGT - gamma glutamyltransferase.					

Table 4 shows the performance characteristics of the parameters performed in a private laboratory in Alexandria governorate. Sigma metrics were calculated using the total allowable errors from the different sources as shown in the table. Among the assayed parameters GGT had the highest sigma (12.8) while calcium had the lowest sigma value (1.0).

**Table 4:** The private laboratory Sigma metrics calculation

**Parameters**	**TEa source**	**TEa**	**Bias (%)**	**CV (%)**	**Sigma**
ALT	CLIA	20.00	1.8	2.9	6.2
Alb	CLIA	10.00	0.3	2.0	4.7
ALP	CLIA	30.00	1.9	6.4	4.4
BD	BV desirable	44.50	0.8	6.4	6.8
BT	CLIA	20.00	2.1	3.5	5.1
Calcium	BV desirable	2.55	1.2	1.4	1.0
Cholesterol	CLIA	10.00	0.0	1.6	6.3
Creatinine	CLIA	15.00	1.3	4.2	3.3
Glucose	CLIA	10.00	2.9	1.8	3.9
LD	CLIA	20.00	3.1	3.4	5.0
Magnesium	CLIA	25.00	1.3	4.2	5.6
Phosphorous	BV desirable	10.10	2.8	1.8	4.1
Total Protein	CLIA	10.00	2.1	1.7	4.6
Triglycerides	CLIA	25.00	0.0	2.0	12.6
Uric Acid	CLIA	17.00	2.0	2.4	6.1
Urea	CLIA	9.00	3.1	2.6	2.3
GGT	BV desirable	22.10	2.9	1.5	12.8
TEa – total allowable error. CV – coefficient of variation. BV – biological variation. CLIA - Clinical Laboratory Improvement Amendments. AFP - alpha fetoprotein. ALT - alanine aminotransferase. Alb – albumin. ALP - alkaline phosphatase. BD – billirubin, direct. BT- bilirubin, total. CEA - carcinoembryonic antigen. LD - lactate dehydrogenase. GGT - gamma glutamyltransferase.					

Table 5 shows comparison between sigma levels after unifying the TEa source for all four laboratories.

**Table 5:** Comparison between different sigma levels after unifying the TEa source

**Parameters**	**TEa source**	**TEa**	**SIGMA MRI**	**SIGMA Zagazig**	**Sigma** **AIN SHAMS**	**Sigma private lab**
AFP	CLIA	31.87	3.4	4.8	--	--
ALT	CLIA	20.00	3.9	2.4	2.1	6.2
Alb	CLIA	10.00	5.1	4.7	0.4	4.7
ALP	CLIA	30.00	4.0	5.5	3.3	4.4
BD	RCPA	20.00	1.9	4.4	1.2	3.0
BT	CLIA	20.00	7.8	4.1	2.7	5.1
Calcium	CLIA	12.20	4.5	5.2	2.0	7.8
CEA	RCPA	12.00	0.9	--	--	--
Cholesterol	CLIA	10.00	1.5	4.3	1.3	6.3
Creatinine	CLIA	15.00	1.3	3.4	1.8	3.3
Glucose	CLIA	10.00	6.3	3.9	4.9	3.9
LD	CLIA	20.00	4.2	5.3	--	5.0
Magnesium	CLIA	25.00	3.5	6.0	2.0	5.6
Phosphorous	RCPA	10.15	1.2	2.2	--	4.1
Total protein	CLIA	10.00	3.0	4.4	1.2	4.6
Triglycerides	CLIA	25.00	7.6	10.3	--	12.6
Uric acid	CLIA	17.00	8.6	6.7	3.0	6.1
Urea	RCPA	21.44	10.3	5.3	2.5	7.1
GGT	RCPA	12.00	0.9	3.0	0.7	6.0
Sigma re-calculated for the four participating laboratories using the same source of TEa. TEa – total allowable error. MRI – medical research institute. CLIA - Clinical Laboratory Improvement Amendments. RCPA - Royal College of Pathologists of Australasia.AFP - alpha fetoprotein. ALT - alanine aminotransferase. Alb – albumin. ALP - alkaline phosphatase. BD – billirubin, direct. BT- bilirubin, total. CEA - carcinoembryonic antigen. LD - lactate dehydrogenase. GGT - gamma glutamyltransferase.						

Tables 6-8 show comparisons of values for bias, CV and sigma between all four laboratories.

**Table 6:** Comparison between bias (%) in different laboratories

**Parameters**	**Medical research institute**	**Zagazig University**	**Ain Shams University**	**The private laboratory**
AFP	9.3	4.2	--	--
ALT	6.7	8.7	5.9	1.8
ALb	0.2	0.6	7.2	0.3
ALp	2.3	2.7	3.9	1.9
BD	5.6	2.7	8.9	0.8
BT	0.0	6.4	2.1	2.1
Calcium	2.0	4.2	0.5	1.2
CEA	1.3	--	--	--
Cholesterol	6.1	3.2	1.7	0.0
Creatinine	5.5	1.9	0.6	1.3
Glucose	1.1	1.7	1.1	2.9
LD	0.5	1.9	--	3.1
Magnesium	10.1	0.5	8.0	1.3
Phosphorous	5.9	1.3	--	2.8
Potassium	2.1	1.1	2.1	--
Total Protein	0.3	1.7	1.4	2.2
Triglycerides	1.6	1.4	--	0.0
Uric Acid	0.7	1.0	3.0	2.0
Urea	1.9	0.0	6.3	3.1
GGT	1.6	2.3	4.6	2.9
AFP - alpha fetoprotein. ALT - alanine aminotransferase. Alb – albumin. ALP - alkaline phosphatase. BD – billirubin, direct. BT- bilirubin, total. CEA - carcinoembryonic antigen. LD - lactate dehydrogenase. GGT - gamma glutamyltransferase.				

**Table 7:** Comparison between CV (%) in different laboratories

**Parameters**	**Medical Research Institute**	**Zagazig University**	**Ain Shams University**	**The private laboratory**
AFP	6.7	5.8	--	--
ALT	3.4	4.6	6.7	2.9
Alb	1.9	2.0	7.2	2.0
ALP	6.9	5.0	8.0	6.4
BD	7.5	3.9	9.4	6.4
BT	2.6	3.3	6.6	3.5
Calcium	2.3	1.9	6.0	1.4
CEA	11.4	--	--	--
Cholesterol	2.6	1.6	6.3	1.6
Creatinine	7.5	3.8	7.9	4.2
Glucose	1.4	2.1	1.8	1.8
LD	4.7	3.4	--	3.4
Magnesium	4.3	4.1	8.4	4.2
Phosphorous	3.5	4.0	--	1.8
Potassium	5.1	2.4	8.0	--
Total protein	3.3	1.9	6.9	1.7
Triglycerides	3.1	2.3	--	2.0
Uric acid	1.9	2.4	4.6	2.5
Urea	1.9	4.0	6.0	2.6
GGT	11.0	3.2	10.4	1.5
CV – coefficient of variation. AFP - alpha fetoprotein. ALT - alanine aminotransferase. Alb – albumin. ALP - alkaline phosphatase. BD – billirubin, direct. BT- bilirubin, total. CEA - carcinoembryonic antigen. LD - lactate dehydrogenase. GGT - gamma glutamyltransferase.				

**Table 8:** Comparison between sigma in different laboratories

**Parameters**	**Medical Research Institute**	**Zagazig University**	**Ain Shams University**	**The private laboratory**
AFP	3.5	3.0	--	--
ALT	6.1	4.1	2.1	6.2
Alb	2.0	4.6	0.4	4.7
ALP	1.4	5.4	3.3	4.4
BD	5.2	10.7	3.8	6.8
BT	10.5	6.2	2.7	5.1
Calcium	0.8	0.7	2.7	1.0
CEA	2.0	--	--	--
Cholesterol	1.1	3.3	1.3	6.3
Creatinine	0.4	1.8	1.8	3.3
Glucose	4.2	3.9	4.9	3.9
LDH	2.3	5.3	--	5.0
Magnesium	- 0.7	1.6	2.0	5.6
Phosphorous	1.2	3.4	--	4.1
Potassium	0.7	1.8	0.8	--
Total protein	1.0	4.3	1.2	4.6
Triglycerides	7.9	10.7	--	12.6
Uric acid	5.9	4.6	3.0	6.1
Urea	7.2	3.9	0.5	2.3
GGT	1.9	6.2	2.1	12.8
AFP - alpha fetoprotein. ALT - alanine aminotransferase. Alb – albumin. ALP - alkaline phosphatase. BD – billirubin, direct. BT- bilirubin, total. CEA - carcinoembryonic antigen. LD - lactate dehydrogenase. GGT - gamma glutamyltransferase.				

**Figure 1:**
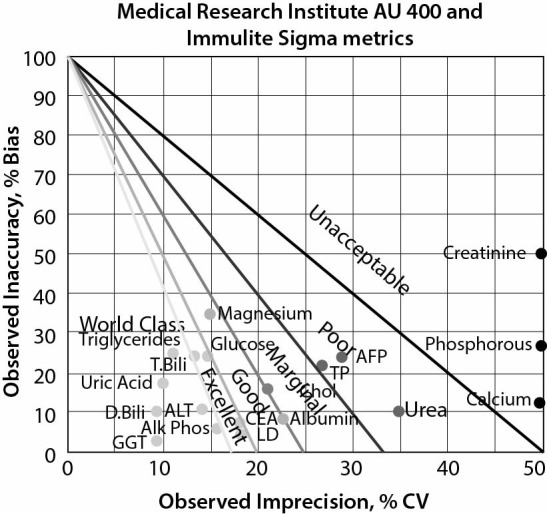
Method decision chart for Medical Research Institute, Alexandria University. D.Bili - direct bilirubin. T.Bili - total bilirubin. TP - total protein. ALT - alanine aminotransferase. Alk Phos - alkaline phosphatase. LD - lactate dehydrogenase. GGT - gamma glutamyltransferase. CEA - carcinoembryonic antigen. AFP - alpha fetoprotein.

**Figure 2:**
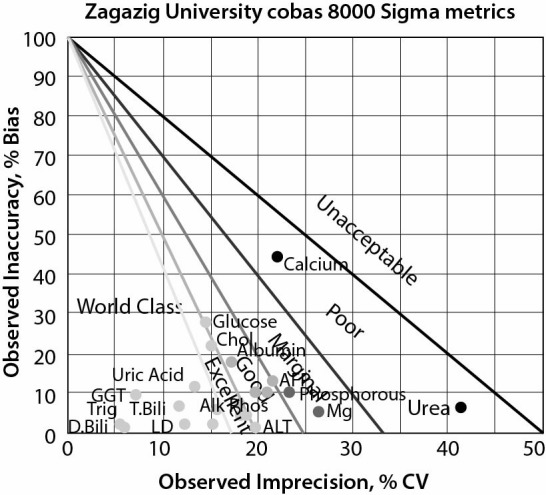
Method decision chart of Zagazig University Hospital laboratory. D.Bili - direct bilirubin. T.Bili - total bilirubin. ALT - alanine aminotransferase. Alk Phos - alkaline phosphatase. LD - lactate dehydrogenase. GGT - gamma glutamyltransferase. AFP - alpha fetoprotein. Trig - triglycerides. Chol - total cholesterol.

**Figure 3:**
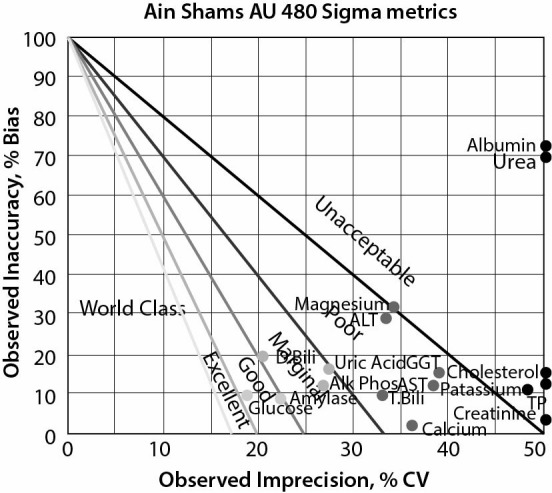
Method decision chart for Ain Shams University hospital laboratory. D.Bili - direct bilirubin. T.Bili - total bilirubin. ALT - alanine aminotransferase. Alk Phos - alkaline phosphatase. TP - total protein. GGT - gamma glutamyltransferase.

**Figure 4:**
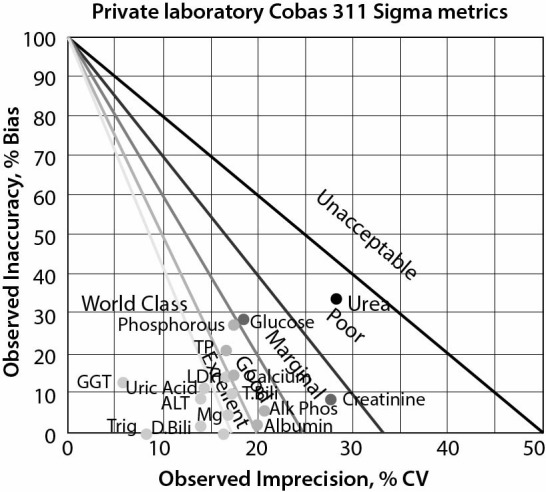
Method decision chart of the private laboratory. D.Bili - direct bilirubin. T.Bili - total bilirubin. ALT - alanine aminotransferase. Alk Phos - alkaline phosphatase. TP - total protein. GGT - gamma glutamyltransferase. LD - lactate dehydrogenase. Mg - magnesium. Trig - triglycerides.

**Figure 5:**
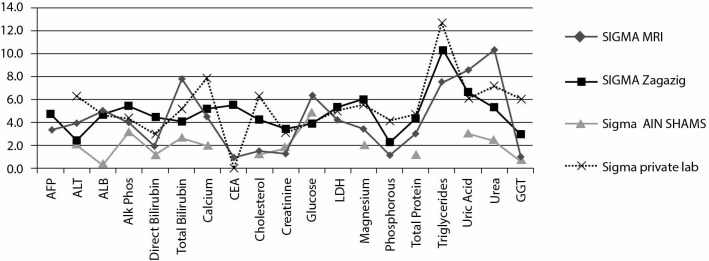
Different Sigma metrics in the four laboratories using the same total allowable error. AFP - alpha fetoprotein. ALT - alanine aminotransferase. Alb – albumin. ALP - alkaline phosphatase. CEA - carcinoembryonic antigen. LD - lactate dehydrogenase. GGT - gamma glutamyltransferase.

Figures 1-4 demonstrate the precision and accuracy of the studied parameters for the four participating laboratories using the method decision chart while Figure 5 shows different sigma metrics in the four laboratories using the same total allowable error.

## Discussion

This study is conducted over one year for four accredited medical laboratories. The sigma values were calculated based on performance approach and were compared to each other in a trial to highlight the lack of objective method of comparability. Control of analytical performance is an essential procedure that shall be done by medical laboratories specially those who are seeking accreditation through method verification which by itself is a standardized process but has no harmonized approach.

Implementing harmonized QC procedures in a medical laboratory requires both knowledge and practical updates. For each of these updates a lot of considerations can be made and a lot of problems can be found. This is the main reason behind lack of harmonized approach in implementing QC procedures. This study is a trial to solve the main problem facing most of the medical laboratories as well as accreditation bodies; which is the lack of harmonized approach for most laboratory`s procedures which results in different healthcare services and outcomes in most laboratories even those which are accreditation by ISO 15189:2012.

Miller and Sandberg recommended that the choice of quality requirements that focus on patient centred care and the optimum clinical decision for each analyte, expressed as TEa based on the change in the analyte would need to be detected in order to make a clinical decision based on that change (*8*). Some analyte changes would affect the clinical decisions when are relatively large (up to 50% for alanine aminotransferase and lipase activities). However, for some of the analytes, a relatively small change will affect the clinical decision in the management of the case such as electrolytes (*8*).

Gami *et al.* studied how different parameters have different biological variation. High biological variation parameter such as triglyceride measured by any instrument will give acceptable sigma level. While electrolytes like sodium and potassium which are having low biological variation would give low results (*15*). In our results the same observation was obtained as well. For triglyceride was calculated by three laboratories very high sigma (Chemical Pathology department Medical Research Institute, Alexandria University, Zagazig University Hospital laboratory and the private lab from Alexandria), while on the other hand potassium that were calculated by three laboratories had very low sigma level (Chemical Pathology department Medical Research Institute, Alexandria University, Zagazig University Hospital laboratory and Ain Shams University hospital laboratory).

The variability of TEa sources as the biological variation data used by Ricos and her colleagues are completely different from the PT limits used by CLIA. Sometimes if we want use the same source like biological variation (BV) the TEa might not be available (*e.g.* direct billirubin) and if available vary due to updating of the studies used and even if all this source of variability are nullified for sake of harmonization. The BV will answer the questions of method performance in three different ways according to which subtype of BV used (optimal TEa which equal half the desirable TEa and one third the minimal TEa).

Precision and bias verification are considered the corner stones of the verification procedures and as mentioned previously both of them have no harmonized protocol, different materials and different targets to achieve, even in case of choosing the same inputs we might get different output for example in case of using the external quality assessment material to determine bias the mean percent bias will be different according to the number, levels, commutability and uncertainty of materials used as all of these characteristics differ from program to another.

Sigma metrics calculation harmonization will help laboratories not to waste time and efforts analysing SM values and changing TEa sources to fit for each analyte. After harmonization the laboratory managers’ efforts will be directed towards the possible causes of poor performance. Taking the calcium in this study as an example, its sigma was unaccepted in three out of four laboratories and this might be due to: improper reagent handling starting from shipment, storage, preparation or on board stability or poor calibration/quality control procedure (reconstitution vehicle, storage and or mixing), or personnel incompetency or lack of instrument preventive maintenance or insufficient environmental conditions monitoring.

After reaching the right root cause, we will have the opportunity to select the proper corrective action and eventually achieve the medical laboratories` ultimate goal which is the high quality patient care.

Comparing the sigma levels in four accredited medical laboratories (three universities and one private laboratory) as an initiative to harmonize the sigma calculations to the best of our knowledge, authors in this study suggested a harmonized protocol for sigma calculation (Annex 1).

Our results showed different sigma levels for different parameters that were calculated using different TEa selected by each laboratory resulting in different categories of performance. This was in agreement with Schoenmakers *et al.* who discussed the variables that affects the sigma calculations and concluded that the use of the road map based on sigma metrics leads to fast and easy implementation of optimal Westgard QC rules. This approach needs standardization in order to lead to better patient care and ultimately in reduction of costs (*16*).

We compared the sigma level of the participating laboratories after unified the selected TEa, most of parameters compared to CLIA TEa except those which had no CLIA limits were compared to RCPA TEa. Table 5 shows SM that was calculated in the four laboratories after harmonization of TEa source. These results highlighted how can the TEa source selection affects the sigma level significantly in a way that may obscure the analytical performance.

Comparing the SM using the same TEa as step towards harmonization gives more real indicator of performance in a more objective approach than using different TEa by each laboratory. For example, total bilirubin SM according to the data calculated by each lab had far worse performance in MRI lab (2.0) than in Zagazig lab (7.8) while after using one common TEa (CLIA) in both laboratories the SM in MRI (7.8) showed better performance than that of Zagazig (4.1). Another example was glucose which according to the data calculated by each lab had almost the same SM in MRI and Zagazig which proved to be wrong when the TEa harmonized in both laboratories (CLIA) and showed that glucose performance at MRI (6.3) was better than that at Zagazig (3.9). Moreover, the data calculated by each laboratory showed that the magnesium had poor performance at MRI and Zagazig laboratory and excellent performance at the private laboratory, but after unifying the source of TEa and recalculating the magnesium SM using the same TEa (CLIA) the performance of magnesium at Zagazig proved to be excellent and even better than that of the private laboratory (6 and 5.6, respectively) and even the magnesium SM at MRI showed some increase (3.5).

The limitation of this study included: the Sigma metric equation as formulated by Westgard (*17*) is a subject of debate, with an alternate calculation being proposed by Coskun *et al.* (*18*, *19*). However, the most commonly used, published, and cited equation remains the Westgard formulation. This Westgard calculation was established through derivation of critical-systematic error equations (*20*) and was confirmed by comparison to CpK (*21*) Finally, numerous official standards committees have accepted the Westgard formulation, notably the International Federation of Clinical Chemistry (IFCC) committee for standardization of HbA1c; they recommend the use of the Westgard Sigma metrics formulation in assessing and selecting HbA1c methods (*22*, *23*). Even the European Federation of Clinical Chemistry and Laboratory Medicine (EFLM) Task and Finish Group on Total Error, while acknowledging the alternate Coskun formulation, accepted the Westgard equation as the standard (*24*). Other EQA programs, such as Dutch Foundation for Quality Assessment in Medical Laboratories (SKML), offer the Westgard Sigma metrics as a standard part of their survey reports (*25*). In 2009, a convocation of experts on quality control issued a collective opinion paper recommending the use of Sigma metrics in the Westgard formulation (*26*). Simply put, the Coskun formulation of the Sigma metrics is neither in wide acceptance nor wide use.

There are many variables that affect the comparability of estimated Sigma metrics among medical laboratories which include: the time interval upon which Sigma metrics is calculated, the different vendor systems providing external proficiency testing programs and quality control programs upon which bias and imprecision values are calculated and different environmental conditions. Also, the variability in the methods used for bias calculation. In addition to the different analytical or clinical benchmarks that are chosen for evaluation of TEa.

The laboratories participating in this study determined analytical bias through EQA programs where the bias was calculated through the difference between laboratory result and that of the EQA group mean against the group mean. Therefore, this is not a true value.

In conclusion, this study is considered the first to highlight the need for Sigma metrics harmonization. Therefore it is mandatory that all laboratory professionals interested in the analytical quality to harmonize the approach of sigma calculation with special empathizes on the bias and CV which are the main components of the sigma equation as well as to unify the methodology used among different laboratories. As for the bias calculation it is recommended to standardize the calculation by using duplicate readings of a number of materials with different concentrations to exclude the element of random error if the PT samples are used as a source for bias calculation. This in turn will help laboratories to find a unified objective tool to judge their method correctly. Finally, the TEa sources shall be vigorously reviewed and only approved sources shall be adopted for calculation. Each laboratory should select the TEa goal based on clear standardized criteria of selection without any subjective preferences as either under or over estimation of Sigma metrics will affect the patient centred care negatively if laboratories use quality control procedures wrongly based on incorrect Sigma metrics calculation with subsequent misleading medical decisions. Laboratories performance using different tolerance limit can’t be compared to each other using sigma approach. Further studies shall be conducted by the accredited laboratories in different sectors adapting the concept of harmonized approach. One of the most important outcomes of this study is the suggested harmonized protocol presented in Annex 1.

## Supplementary material

Annex1a.

Annex1b.
