# Supplementary figures and images for: Comparison between Sigma metrics in four accredited Egyptian medical laboratories in some biochemical tests: an initiative towards sigma calculation harmonization
Source: Biochem Med (Zagreb). 2018 Jun 15;28(2):020711. doi: 10.11613/BM.2018.020711 (PMC6039160; doi:10.11613/BM.2018.020711)

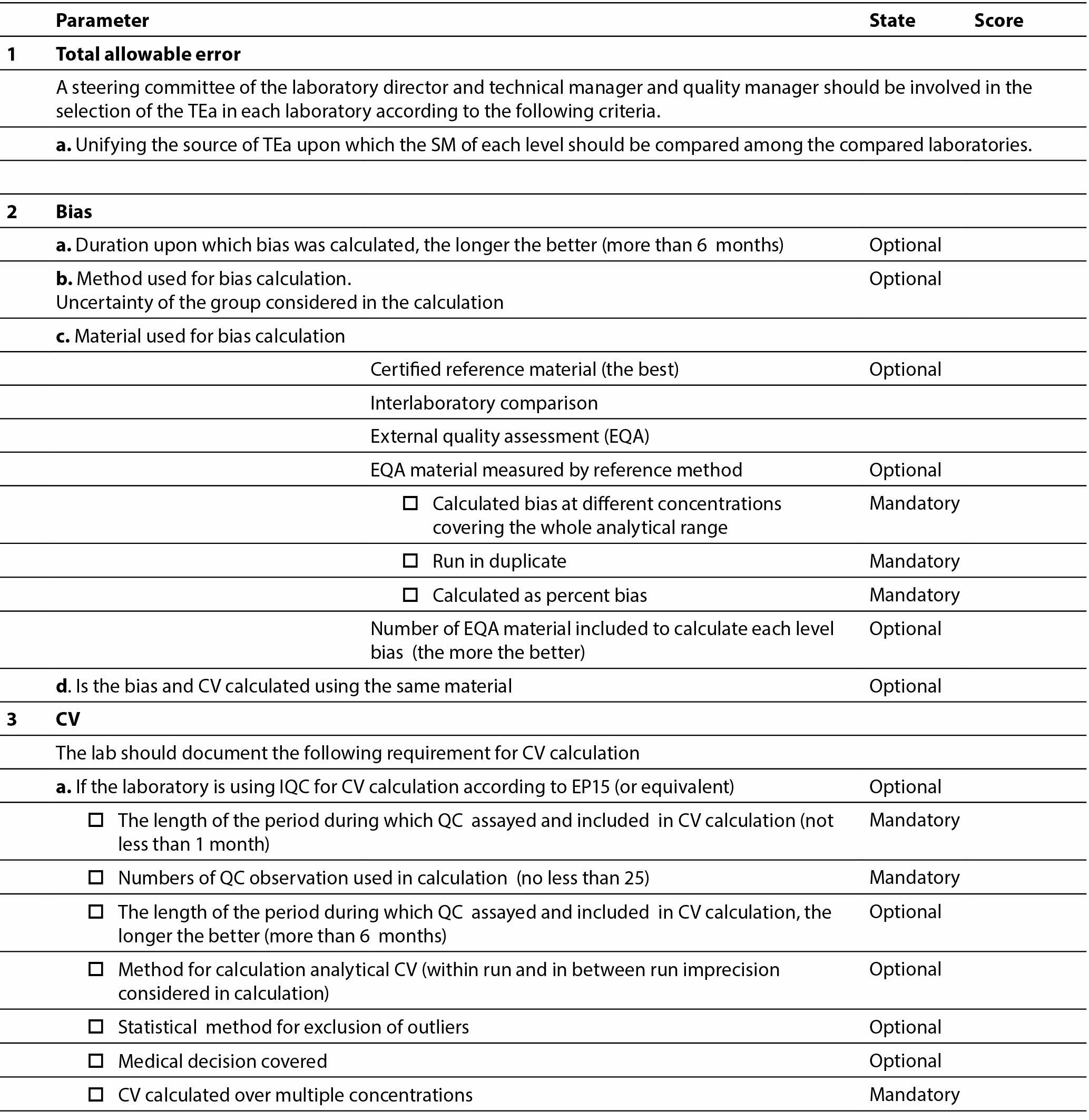

Supplement: Supplementary file 1 — Annex1a. [file bm-28-2-020711-S2.tif]
